# Cardiogenic Shock: Clinical Management, Outcomes and Future Directions

**DOI:** 10.3390/jcdd13040156

**Published:** 2026-03-31

**Authors:** Aaqil Ahmad Aubdool, Andrew J. Sullivan, Daniel A. Jones, Anthony Mathur, Alastair Proudfoot, Krishnaraj S. Rathod

**Affiliations:** 1 Barts and The London School of Medicine and Dentistry, Queen Mary University of London, London E1 2AD, UK; 2 Barts Heart Centre, St. Bartholomew’s Hospital, London EC1A 7BE, UKdan.jones8@nhs.net (D.A.J.);; 3 Centre for Cardiovascular Medicine and Devices, William Harvey Research Institute, Queen Mary University of London, London E1 4NS, UK; 4 NIHR Barts Biomedical Research Centre, Queen Mary University of London, Charterhouse Square, London EC1M 6BQ, UK

**Keywords:** cardiogenic shock, intra-aortic balloon pump (IABP), Impella, extracorporeal membrane oxygenation (ECMO), percutaneous coronary intervention (PCI), coronary artery bypass graft (CABG)

## Abstract

Cardiogenic shock is a life-threatening condition caused by the heart’s sudden inability to pump sufficient blood to maintain adequate tissue perfusion, most commonly occurring following a myocardial infarction or acute decompensated heart failure. The resultant hypoperfusion can quickly progress to end-organ failure and ultimately death if not treated urgently. This review explores the management of cardiogenic shock, highlighting current treatments, their effectiveness, and the challenges faced by healthcare providers. It looks at both pharmacological therapies and devices used for cardiac support, including mechanical circulatory support and emergency revascularisation procedures to restore blood flow. We also examine how different stages of shock affect survival and how new technologies including artificial intelligence and wearable monitors could help detect and treat this condition earlier. In addition, this review discusses the significant pressure that cardiogenic shock places on healthcare provision, including the typical financial cost of treatment in the UK, resource utilisation and regional disparities. Finally, we outline future directions for trial design, better prevention, more rapid diagnosis and improved treatments that could improve morbidity and mortality.

## 1. Introduction

Cardiogenic shock (CS) is a severe and life-threatening condition characterised by an acute impairment in heart contractility, leading to reduced cardiac output (CO), poor end-organ perfusion and the need for rapid intervention [1,2,3]. Most commonly occurring as a complication of acute myocardial infarction (AMI), CS remains a critical clinical emergency in cardiology with persistently high in-hospital and long-term mortality rates, despite significant advances in pharmacological therapy and interventional cardiology.

In the UK and globally, CS continues to pose substantial challenges to healthcare systems due to its complex pathophysiology, heterogeneity in presentation, and the urgency of diagnosis and treatment. Landmark trials such as the SHOCK (SHould we emergently revascularize Occluded Coronaries for cardiogenic shock) trial and more recent data from the CULPRIT-SHOCK, IABP-SHOCK II and DanGer-SHOCK studies have shaped contemporary understanding and management of AMI-associated CS (AMI-CS) [4,5,6]. However, there remains a considerable evidence gap in the optimal use of mechanical circulatory support (MCS) devices and the management of non-AMI causes of CS, such as acute decompensated heart failure (HF) and myocarditis.

The implementation of standardised clinical definitions and staging systems, most notably the Society for Cardiovascular Angiography and Interventions (SCAI) classification, has improved the clinical stratification of CS and facilitated earlier recognition [7]. Yet, significant variability persists in clinical practice within the UK due to the lack of a formal, national network of care for CS or pathway for CS patients.

Moreover, the economic burden of CS on healthcare providers is substantial, driven by prolonged intensive care admissions, high costs of advanced cardiac interventions, and long-term rehabilitation needs. In parallel, emerging technologies—including biomarker-driven phenotyping, artificial intelligence (AI), and smart wearables—offer new opportunities for earlier detection, improved risk stratification, and individualised management, but their integration into routine care remains limited and requires further validation.

This review aims to critically evaluate the current management strategies for CS in the UK, with a particular focus on the clinical efficacy of existing interventions, the burden on healthcare resources, and the potential of emerging innovations to improve patient outcomes. By synthesising evidence from recent trials, national guidelines, and health service data, the project seeks to identify areas of unmet need and propose future directions for clinical practice and policy development.

## 2. Background

### 2.1. Definition

The Heart Failure Association of the European Society of Cardiology defines CS as “a syndrome caused by a primary cardiovascular disorder in which inadequate cardiac output results in a life-threatening state of tissue hypoperfusion associated with impairment of tissue oxygen metabolism and hyperlactatemia which may result in multiorgan dysfunction and death” [2]. Over time, the clinical definition of CS has evolved across guidelines and large studies, with landmark trials and contemporary guidelines offering refined criteria [8]. Recognising the need for greater precision and standardisation, the Shock Academic Research Consortium (SHARC) proposed updated consensus definitions to harmonise clinical practice and clinical research [9].

- Definition for clinical practice: “Cardiac disorder that results in both clinical and biochemical evidence of sustained tissue hypoperfusion.”
- Definition for clinical trials: “Cardiac disorder that results in a systolic blood pressure < 90 mm Hg for ≥30 min (or the need for vasopressors, inotropes or mechanical circulatory support to maintain systolic blood pressure ≥ 90 mm Hg) with evidence of hypoperfusion.”

Evidence of hypoperfusion includes clinical features (cool or mottled extremities, altered mental status not explained by an alternative cause) and/or biochemical and organ-specific markers, namely acute hepatic injury (e.g., ALT > 3 times upper limit of normal), acute kidney injury (creatinine ≥ 2 times upper limit of normal) or oliguria (urine output < 0.5 mL/(kg/h), or elevated arterial lactate > 2 mmol/L. Importantly, SHARC definitions recognise that CS may occur in the absence of sustained hypotension, provided there is evidence of hypoperfusion or the need for vasoactive support.

In contrast, the FRENSHOCK registry adopts a pragmatic, real-world framework based on observational practice [10]. FRENSHOCK defines CS by the presence of haemodynamic impairment, left and/or right cardiac overload, and organ hypoperfusion, requiring at least one criterion from each domain. By explicitly incorporating congestion and hypoperfusion phenotypes, the FRENSHOCK registry captures a broad spectrum of patients and reflects the heterogeneity of CS encountered in the clinical setting. These definitions allow pragmatic recognition of CS in practice, while providing strict and reproducible criteria for inclusion in clinical trials.

### 2.2. Classification

More recently, the SCAI introduced a standardised classification system that categorises CS into five progressive stages (A to E) [7]. Stage A represents a patient “at risk” for CS without overt haemodynamic instability, while Stage B (“beginning shock”) indicates early signs of hypotension without hypoperfusion. Stage C (“classic shock”) defines the traditional picture of CS with hypotension and evidence of hypoperfusion requiring intervention. Stage D (“deteriorating shock”) reflects failure to respond to initial treatment, and Stage E (“extremis”) describes refractory shock with imminent risk of death despite maximal support.

The SCAI Shock Classification provides a valuable framework for early recognition, risk stratification, and timely intervention of CS. The updated classification from 2021 further emphasised not only the spectrum of severity within stages but also the importance of modifiers (e.g., cardiac arrest, ventilatory support, renal dysfunction, lactate levels) that refine prognostic estimates [7]. Identifying patients in the earlier stages (A or B) allows for preventive measures to halt the progression to more severe stages, while early intervention in later stages (C–E) can significantly improve outcomes by preserving organ perfusion and reducing mortality. Validation studies have shown a clear correlation between higher SCAI stages and increased mortality, with prognosis differing substantially across stages [11,12]. For instance, Stage E patients often face high mortality rates despite aggressive interventions such as mechanical circulatory support, underscoring the importance of early identification and treatment of at-risk patients [12].

Studies from the Critical Care Cardiology Trials Network (CCCTN) and the Cardiogenic Shock Working Group (CSWG) have demonstrated that the SCAI stage is dynamic—serial reassessment, particularly in the first 24 h, provides strong prognostic value. For example, Ton et al. in the CSWG registry (*n* = 3268; ~57% HF-CS, 27% MI-CS) showed that more than half of patients in Stages B or C worsened by 24 h, and those who deteriorated (or who remained at/progressed to Stage E) had the worst prognosis [13]. Similarly, in a large ICU cohort (*n* = 21,461), Jentzer et al. showed that serial determination of SCAI stage (in 4 h blocks over the first 24 h of admission) led to better discrimination of in-hospital mortality than a single admission SCAI stage [14]. The SCAI classification with dynamic reassessment thus serves as a critical tool for clinicians to optimise care and improve outcomes through enabling early and repeatedly updated risk stratification and more tailored therapeutic decisions in this challenging condition.

### 2.3. Pathophysiology

**Figure 1:**
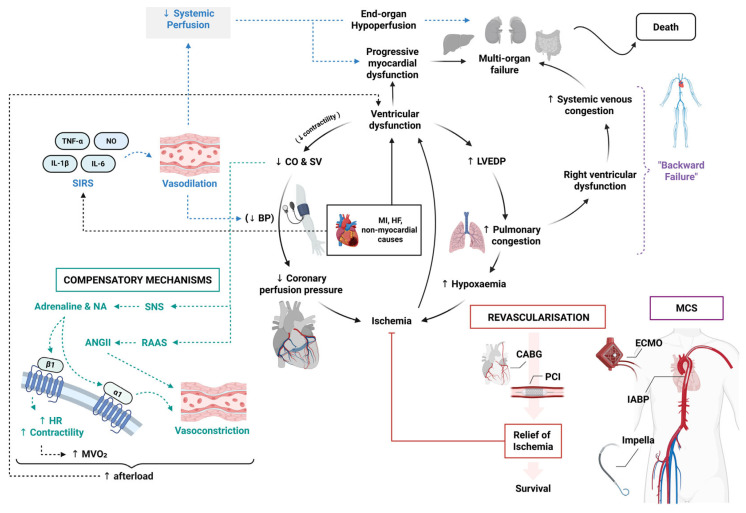
The pathophysiology of cardiogenic shock (CS); the cycle of worsening ventricular dysfunction leading to multi-organ failure. Compensatory mechanisms via SNS and RAAS activation aim to maintain cardiac output and restore perfusion while increasing afterload and furthering haemodynamic decline. Revascularization provides relief of ischemia and improves survival outcomes while MCS acts as a bridge to more definitive therapy. MI (myocardial infarction). HF (heart failure). LVEDP (left ventricular end-diastolic pressure). BP (blood pressure). CO (cardiac output). SV (stroke volume). SIRS (system inflammatory response syndrome). TNF-α (tumour necrosis factor-alpha). IL-6 (interleukin-6). IL-1β (interleukin-1β). NO (nitric oxide). SNS (sympathetic nervous system). NA (noradrenaline). RAAS (renin–angiotensin–aldosterone system). ANGII (angiotensin II). HR (heart rate). MVO_2_ (myocardial oxygen demand). MCS (mechanical circulatory support). CABG (coronary artery bypass graft). PCI (percutaneous coronary intervention). ECMO (extracorporeal membrane oxygenation). IABP (intra-aortic balloon pump). Created in BioRender. Aubdool, A. (2026). https://BioRender.com/umhprqe (accessed on 15 March 2026).

The pathophysiology of CS varies according to the underlying aetiology and is most often driven by impaired ventricular function (left, right or biventricular failure) which can affect systolic or diastolic function; however, even moderate dysfunction of the left or right ventricle may be sufficient to precipitate CS in the setting of acute heart failure, right ventricular failure (e.g., pulmonary embolism), arrhythmias or valvular pathology [3]. The severe reduction in myocardial contractility results in a decrease in CO and stroke volume (SV) (Figure 1) [1,2,15]. This leads to a characteristic decline in coronary perfusion pressure, further impairing myocardial oxygen delivery and initiating an overall cycle of ischemia and worsening myocardial dysfunction. The ischemic myocardium contributes to elevated left ventricular end-diastolic pressure (LVEDP), leading to pulmonary congestion and hypoxemia, worsening tissue hypoxia, exacerbating cellular injury and triggering metabolic disturbances.

A critical aspect of CS pathophysiology is the activation of compensatory mechanisms, particularly sympathetic nervous system (SNS) activation and renin–angiotensin–aldosterone system (RAAS) upregulation (Figure 1) [16]. SNS activation triggers the release of adrenaline and noradrenaline (NA) [3,15]. This increases heart rate (HR) and contractility in an attempt to maintain CO and restore perfusion. However, this compensatory response also elevates myocardial oxygen demand (MVO_2_) and causes intense vasoconstriction, increasing afterload and perpetuating a vicious cycle of worsening ventricular dysfunction and haemodynamic collapse [16]. Over time, excessive β1-adrenergic stimulation leads to myocardial calcium overload, promoting arrhythmias and cardiomyocyte apoptosis, while α1-mediated vasoconstriction exacerbates end-organ hypoperfusion.

Simultaneously, the RAAS is activated, increasing angiotensin II and aldosterone levels [17]. While this initially promotes vasoconstriction and sodium retention to maintain BP, it also increases afterload, further worsening left ventricular failure and coronary hypoperfusion.

Beyond haemodynamic disturbances, a growing body of evidence highlights the pivotal role of dysregulated inflammation in CS [18,19]. Systemic inflammatory response syndrome (SIRS) is triggered where pro-inflammatory cytokines, including tumour necrosis factor-alpha (TNF-α), interleukin-6 (IL-6), and interleukin-1β (IL-1β), are released in response to SNS activation, ischemia–reperfusion injury, and cellular stress [19,20,21]. These mediators, along with nitric oxide (NO) and peroxynitrate which can have cardiotoxic effects, promote peripheral vasodilation, leading to worsening hypotension and perfusion deficits [3]. Leukocyte infiltration further contributes to CS, which studies have shown to be statistically significant in increasing 30-day mortality [22,23]. This inflammation-driven microvascular dysfunction disrupts coronary autoregulation, exacerbating myocardial ischemia even when large coronary arteries are revascularised.

In addition to the left ventricle, right ventricular failure, particularly in inferior MI or pulmonary hypertension, further contributes to CS via increased pulmonary congestion, reducing left ventricular preload and further worsening systemic hypoperfusion [20]. Impaired right ventricular function also results in systemic venous congestion, termed “backward failure”, contributing to multi-organ dysfunction, particularly in the kidneys, liver, and intestines. Additionally, tissue hypoxia leads to anaerobic metabolism and metabolic acidosis, which further impairs cardiac contractility and vascular responsiveness, worsening haemodynamic collapse.

### 2.4. Aetiology

CS arises from a spectrum of underlying cardiovascular disorders with AMI historically being recognised as one of the leading causes of CS. A total of 7–10% of patients presenting with AMI deteriorate and go on to develop CS [24]. In these patients, extensive acute cardiac ischemia results in a rapid impairment in contractility and subsequent reduced CO and tissue hypoperfusion. Alongside impaired contractility found in AMI, the mechanical complications such as ventricular septal defect (VSD), free wall rupture, or papillary muscle rupture may also precipitate CS as shown in the SHOCK trial where these were shown to account for 12% of CS cases [4,25,26].

While AMI remains a common cause of CS, contemporary cohorts, particularly in tertiary and quaternary centres, demonstrate a substantial and increasing burden of non-ischaemic aetiologies, including pulmonary embolism, myocarditis, cardiomyopathies, pregnancy and post-cardiotomy shock. Contemporary registry data suggest that the epidemiology of CS is evolving and AMI-CS cases are outnumbered in many studies. Analysis from the CCCTN found that, among 8974 patients admitted to advanced cardiac intensive care units between 2017 and 2023, heart failure-related CS (HF-CS) was the most frequent aetiology, comprising ~59% of isolated CS cases [27]. Within this group, 72% represented acute-on-chronic decompensation and 28% de novo presentations while AMI-CS accounted for 27% of cases, two-thirds of which were STEMI.

Beyond AMI and HF, other secondary (non-myocardial) causes of CS include severe valvular disease and cardiac arrhythmias which also disrupt effective CO [28,29]. Massive pulmonary embolism is a recognised aetiology of CS with significant 30-day mortality, where haemodynamic compromise may occur even in the absence of hypotension [30]. Post-cardiotomy CS (PCS) is a less studied and rare yet increasingly prevalent cause of CS, which occurs following failure to wean from cardiopulmonary bypass, seen in 0.5–1.5% of postoperative cardiac surgery patients [31]. Non-ischemic or genetic cardiomyopathies, including dilated [32], restrictive [33], inflammatory forms such as myocarditis, and stress-induced (Takotsubo) cardiomyopathy, represent important causes of CS, where acute deterioration in myocardial function may lead to rapid haemodynamic compromise [32,34]. Pregnancy-associated CS may arise from a range of medical or obstetric-specific precipitants, including amniotic fluid embolism and peripartum cardiomyopathy; although uncommon, as a decompensation of cardiac disease in pregnancy, it represents a major contributor to maternal mortality [35]. These non-AMI causes, although less frequently captured in randomised controlled trials (RCTs), underscore the heterogeneity of CS and the need for tailored management strategies.

### 2.5. Risk Factors

Several factors increase the risk of developing CS and poorer prognostic outcomes such as old age, sex, comorbidity, multi-vessel coronary artery disease (CAD), complete atrio-ventricular (AV) block, previous MI or HF and anterior ST-segment elevation MI (STEMI) [25].

Old age is a major risk factor for a CS due to a higher burden of comorbidities and reduced cardiac reserve. A study conducted across three registries in France illustrated that patients ≥ 75 years are more vulnerable to developing CS when compared to younger age groups [36,37]. Furthermore, with advancing age, the patient’s ability to withstand the stress associated with CS is greatly diminished, increasing the mortality risk, particularly in the later SCAI stages of CS [38].

Sex differences also play a role where women are at higher risk for poorer outcomes, partly due to delayed diagnoses and atypical presentations contributing to worse prognoses. A report from the SHOCK trial registry highlighted that while there was a lack of correlation between sex and in-hospital mortality, more women developed mechanical complications such as ventricular septal rupture (VSR) (7.7% vs. 3.5%, *p* = 0.003) and severe mitral regurgitation (MR) (11.4% vs. 7.1%, *p* = 0.014) and women more often required blood transfusions compared to men (47% vs. 35%, *p* = 0.001) [39]. Interestingly, as concluded by a systematic review of patient characteristics in 14 AMI-CS RCTs (N = 2154) compared to 12 registries (N = 133,617), RCTs enrolled fewer women (27.0% vs. 32.3%) compared with real-world patients in registries, highlighting the need for improvements in the inclusion of women in such trials to better inform clinical practice [40].

Chronic hypertension and diabetes mellitus are contributors to the development of multi-vessel CAD and endothelial dysfunction, both of which predispose individuals to CS. Contrary to the results of the SHOCK trial which only showed a marginally lower survival rate in diabetics [41], a systematic review and meta-analysis on the impact of diabetes on CS outcomes demonstrated that diabetes was an adverse prognostic indicator in AMI-CS, with patients having an increased risk of in-hospital mortality, major bleeding, reinfarction, revascularisation necessity, cerebrovascular events and length of hospital stay [42]. Diabetes is therefore not only a key risk factor for CS but also appears to confer increased morbidity and mortality.

Multi-vessel CAD further exacerbates the risk of CS as it reduces the heart’s ability to compensate for additional stress, particularly in older patients or those with pre-existing conditions [25]. Complete AV block, especially in the context of MI, can lead to inadequate CO and CS due to slow ventricular rates. A history of previous MI or HF, particularly with left ventricular dysfunction, is a major predictor of CS risk [43]. Additionally, anterior STEMI, affecting the left ventricle, is a high-risk condition for shock due to the large myocardial area involved, which can lead to significant cardiac dysfunction.

## 3. Current Treatment Approaches and Clinical Guidelines

### 3.1. Guidelines

In the UK, the management of CS is guided by a combination of NICE (National Institute for Health and Care Excellence) recommendations and international guidelines. A pivotal document is the “Shock to Survival” framework published by the Intensive Care Society, which emphasises the critical importance of early recognition, assessment, and treatment of CS [44]. This framework acknowledges the variability in healthcare services and clinical pathways across the UK and advocates for rapid decision-making to improve patient outcomes. Additionally, the European Society of Cardiology (ESC) provides comprehensive guidelines [45]. These guidelines underscore the necessity of a structured and timely approach to CS management, aiming to reduce the persistently high mortality rates associated with the condition.

### 3.2. Current Management

Management of CS requires a multifaceted approach aimed at restoring adequate CO and perfusion while addressing the underlying cause. Current strategies include pharmacological treatments, MCS devices, and timely revascularisation, all guided by clinical protocols and evidence from landmark trials.

#### 3.2.1. Pharmacological Therapy

Pharmacological therapy consists of vasopressors and inotropes which are utilised in the initial stabilisation of CS, supporting systemic perfusion and cardiac function while bridging to MCS and more definitive therapies such as revascularisation or durable ventricular assist device (VAD) and heart transplant where indicated [3]. European guidance generally endorses the use of NA or dobutamine as first-line vasoactive medications in CS [46].

##### Vasopressors

Vasopressors such as NA, adrenaline and dopamine are used to increase systemic vascular resistance and maintain BP in hypotensive patients [46]. NA works by stimulating α1-adrenergic receptors to induce vasoconstriction and β1-adrenergic receptors to support CO. NA is indicated in patients with profound hypotension (e.g., SBP < 70 mmHg) or those showing signs of end-organ hypoperfusion despite adequate fluid resuscitation [3].

In addition to catecholamines, non-adrenergic vasopressors such as vasopressin and angiotensin II may have a role in selected patients with CS, particularly those with refractory hypotension despite optimisation of first-line agents. Vasopressin acts on V_1a_ receptors on vascular smooth muscle to induce systemic vasoconstriction without direct β1 stimulation [46] and relative pulmonary vasopressor-sparing effects, thus favouring its use in right ventricular failure [47,48,49]. Vasopressin can help reduce catecholamine requirements and has been associated with a reduced risk of arrhythmia in comparison to catecholamines [50]. Angiotensin II, acting via the RAAS, also produces potent vasoconstriction; data suggest that it can increase mean arterial pressure (MAP) and reduce other vasopressor dosage requirements [51]. While robust RCT data in pure CS remain limited, these agents are increasingly considered as adjunctive therapy in refractory shock.

##### Inotropes

Inotropes are a key component of CS management which target improving perfusion by increasing CO through increasing myocardial contractility. For example, adrenaline (epinephrine) is a catecholamine inotropic agent indicated in hypotensive CS patients predominantly exhibiting β-adrenergic effects. At low doses (typically <0.05 μg/kg/min) it acts primarily as an inotrope to increase CO, whereas α-adrenergic vasoconstrictive effects become more prominent at higher doses, contributing to its vasopressor activity in hypotensive CS. Dobutamine, another widely used inotrope, acts predominantly on β1-adrenergic receptors, both increasing myocardial contractility and subsequently restoring CO [3]. It is typically indicated in CS patients with low CO and evidence of severe tissue hypoperfusion despite adequate BP. Milrinone, a phosphodiesterase-3 inhibitor, is another inotrope with similar pharmacodynamic characteristics to dobutamine indicated in normotensive CS cases such as decompensated HF-CS [46]. Dobutamine and milrinone are often compared in efficacy and safety [52] and the recent DOREMI (dobutamine compared with milrinone) RCT in CS showed neutral results in primary and secondary outcomes with no significant differences between the two agents [53]. Given these findings, dobutamine or milrinone may be used as first-line agents in normotensive CS patients with low CO; however, in the presence of severe renal impairment, dobutamine is the preferred choice. Another available inotropic agent is levosimendan, a calcium sensitiser, with similar haemodynamic effects to milrinone. Similarly though, comparisons to dobutamine have not demonstrated superiority [54,55].

While these agents may be effective for initial haemodynamic stabilisation, their prolonged use can exacerbate myocardial ischemia and arrhythmias, necessitating careful monitoring and early transition to definitive treatments [56,57]. The choice and sequencing of vasoactive agents may differ in non-AMI CS, particularly in right ventricular failure (e.g., pulmonary embolism) or cardiomyopathies, where excessive increases in afterload or myocardial oxygen demand may be poorly tolerated and vasoactive therapy needs to be carefully tailored [58].

#### 3.2.2. Mechanical Circulatory Support (MCS)

MCS devices are critical for patients with refractory CS who do not respond adequately to pharmacological therapy [3]. These devices provide haemodynamic support, reduce myocardial workload, and maintain perfusion to vital organs, buying time for myocardial recovery or more definitive interventions such as revascularisation and transplantation. Consideration and selection for MCS involves evaluation by an experienced interprofessional team.

##### Intra-Aortic Balloon Pump (IABP)

**Figure 2:**
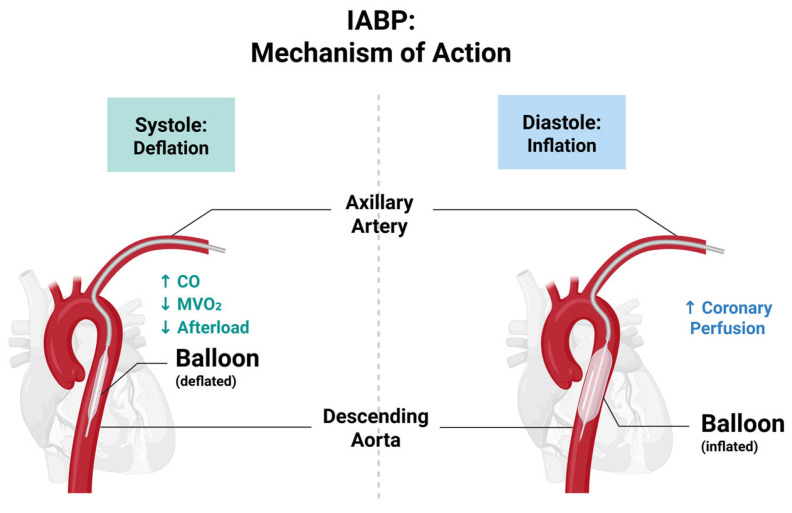
Mechanism of action of the IABP. IABP (intra-aortic balloon pump). CO (cardiac output). MVO_2_ (myocardial oxygen demand). Created in BioRender. Aubdool, A. (2026). https://BioRender.com/umhprqe (accessed on 15 March 2026).

Intra-Aortic Balloon Pump (IABP) is one method of MCS that has long been considered for patients with AMI-CS, severe MR, or VSR [59,60]. IABP works via the inflation of a balloon in the descending aorta during diastole, increasing coronary perfusion, and deflation during systole to reduce afterload and MVO_2_ (Figure 2) [60]. The device thus provides modest haemodynamic support, improving CO and reducing ischemic burden.

However, the role of the IABP, particularly in AMI-CS cases, has come into question in recent years following the results of the IABP-SHOCK II trial, which showed no survival benefit of IABP in AMI-CS [5]. This landmark study, published in The New England Journal of Medicine in 2012, was a randomised, multicentre trial that evaluated the efficacy of IABP in AMI-CS patients undergoing early revascularisation, primarily via percutaneous coronary intervention (PCI) [5]. The trial found no significant difference in 30-day all-cause mortality between patients treated with IABP and those without IABP support. The mortality rate was 39.7% in the IABP group compared to 41.3% in the control group (*p* = 0.69). There were also no significant differences in secondary endpoints, including time to haemodynamic stabilisation, length of stay in the intensive care unit (ICU), serum lactate levels (a marker of tissue perfusion) and rates of recurrent MI, stroke, or major bleeding. A follow-up study at 6 and 12 months confirmed the initial findings, showing no mortality benefit with IABP use. The 12-month mortality rates were 52% in the IABP group and 51% in the control group (*p* = 0.91) [61]. More recently in 2019, IABP was shown to have no effect on mortality at long-term 6-year follow up [62].

The results of the IABP-SHOCK II trial led to a downgrade in the recommendation for IABP use in AMI-CS in major clinical guidelines. For example, the ESC guidelines now recommend against the routine use of IABP in AMI-CS (Class III recommendation) [63] and the American Heart Association (AHA) guidelines also reflect this change, suggesting that IABP should not be routinely used unless there is a specific indication, such as mechanical complications of AMI (e.g., acute MR or VSR) [64]. As a result, there has been a shift toward alternative MCS devices, such as ECMO and microaxial flow devices, e.g., Impella, which provide more robust haemodynamic support [65]. However, evidence for these devices is still evolving, and their use is often limited by availability and expertise.

Recent randomised data and meta-analysis indicate that IABP does not uniformly improve outcomes in HF-CS when added early to standard care [66,67]. In the Altshock-2 trial, early IABP yielded no significant benefit in 60-day survival or successful bridging to heart replacement therapies compared to standard care in patients across SCAI shock stages B–D [67]. Meta-analytic pooling (Baldetti et al.) suggests that benefit, if present, is likely confined to patients in more severe HF-CS (SCAI C/D), rather than those in milder stages [66]. These findings underscore the importance of careful patient selection: in severe HF-CS patients with potential for recovery or transplantation/bridge-to-therapy, IABP remains an option, but its routine use across all HF-CS is not exclusively supported by the current evidence.

Although routine use of IABP has not demonstrated a mortality benefit in CS populations, there are specific clinical scenarios in which IABP may prove to be of haemodynamic advantage. In acute CS due to acute MR or VSD, IABP may be of benefit. In a retrospective cohort of patients with post-AMI VSD or acute MR and CS, IABP support was associated with lower 30-day mortality and acted as an effective bridge to definitive therapy such as surgical or transcatheter mitral valve intervention [59].

##### Extracorporeal Membrane Oxygenation (ECMO)

**Figure 3:**
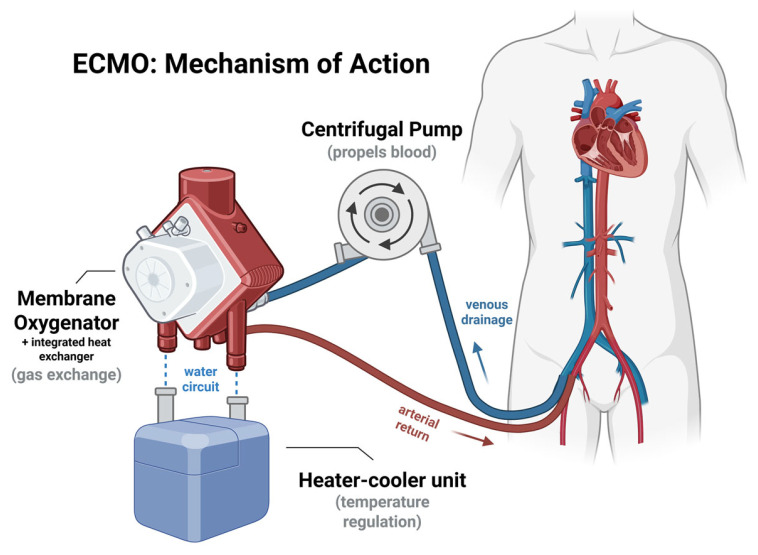
Mechanism of action of VA-ECMO. Venous blood is drained via a large-bore cannula and propelled by a centrifugal pump through a membrane oxygenator, where oxygenation and carbon dioxide removal occur. The membrane oxygenator incorporates an integrated heat exchanger, which is connected to an external heater–cooler unit via a separate water circuit (no blood flow), allowing active warming or cooling as clinically indicated. Oxygenated blood is returned to the arterial circulation, providing systemic perfusion and oxygen delivery independent of native cardiac output. Created in BioRender. Aubdool, A. (2026). https://BioRender.com/umhprqe (accessed on 15 March 2026).

ECMO is another form of MCS indicated for patients with severe CS and impending multi-organ failure, particularly when there is reversible myocardial dysfunction or as a bridge to more definitive therapies such as heart transplantation or ventricular assist devices [68]. Candidates are typically assessed for the absence of irreversible comorbidities, such as advanced malignancy, severe neurological damage, or severe end-stage organ failure. VA-ECMO (veno-arterial ECMO) provides extracorporeal circulation, oxygenating venous blood and removing carbon dioxide while bypassing the failing heart and lungs (Figure 3). VA-ECMO is commonly used in CS to restore perfusion and improve oxygenation, though it increases left ventricular afterload and often necessitates additional unloading measures [69].

Recent evidence has challenged the routine use of ECMO in patients with infarct-related CS, in multicentre RCT which enrolled 420 AMI-CS patients [70]. Participants were randomly assigned to receive either early ECMO plus standard care or standard care alone. The primary outcome, all-cause mortality at 30 days, occurred in 47.8% of patients in the ECMO group and 49.0% in the control group, showing no statistically significant mortality benefit with the early use of ECMO (relative risk 0.98; 95% CI, 0.80 to 1.19; *p* = 0.81). Additionally, ECMO was associated with higher rates of complications such as bleeding and peripheral ischemia.

These findings suggest that while ECMO may offer physiological support, it does not confer a short-term survival advantage and may expose patients to additional risks. However, the study had some limitations, including the exclusion of patients with severe hypoxaemia or cardiac arrest requiring prolonged resuscitation, which may limit generalisability to the sickest subgroup [70]. Furthermore, crossovers between groups and challenges in standardising post-randomisation care may have influenced outcomes. Despite these limitations, the trial provides robust evidence that early ECMO should not be routinely implemented in all patients with infarct-related CS, highlighting the need for more selective, personalised approaches and further research into optimal patient selection.

The ECLS-SHOCK trial at 1-year follow up reaffirmed that no new randomised data overturned its neutral findings, with routine early VA-ECMO in AMI-CS failing to show mortality benefit compared with standard care [71]. These results also emphasised avoiding reflex early VA-ECMO and considering selective use, such as in those with refractory hypoxaemia, severe right ventricular failure, or as a bridge within structured shock pathways.

Although much of the evidence base for ECMO derives from AMI-CS, a substantial and growing population of patients requiring ECMO present with non-ischemic aetiologies. Massive pulmonary embolism (MPE)-induced refractory CS is a potential indication for ECMO and is a Class IIb recommendation in ESC guidelines where ECMO may be considered as an adjunct to open surgical embolectomy or percutaneous catheter-based intervention if the patient meets specific ECMO placement criteria [72]. However, the evidence is limited and heterogeneous, with registry data suggesting possible mortality benefit when combined with reperfusion, but the meta-analyses show no clear survival advantage of ECMO alone versus conventional management, and its use is associated with significant multisystem morbidity that must be weighed in patient selection [30].

##### Microaxial Flow Pumps

**Figure 4:**
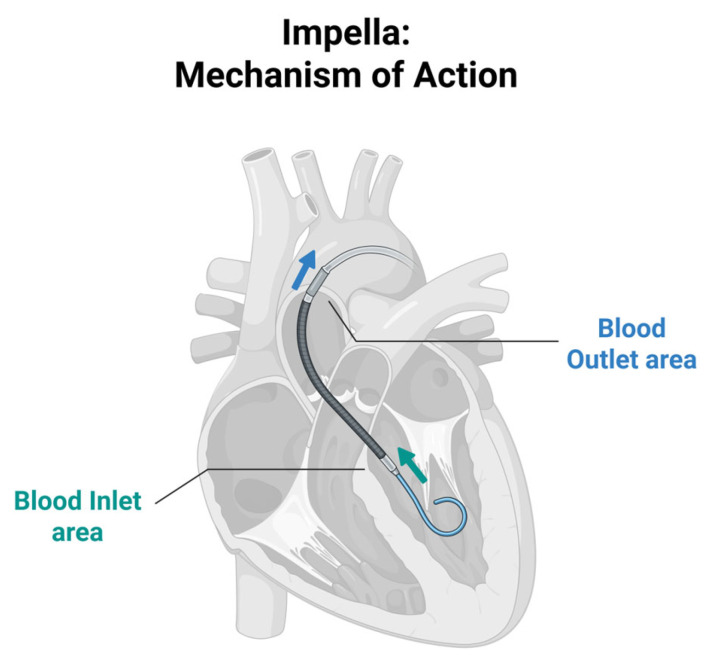
Mechanism of action of Impella includes a catheter-mounted axial flow pump that draws blood from the left ventricle via the inlet area and ejects blood into the ascending aorta via the outlet area. Created in BioRender. Aubdool, A. (2026). https://BioRender.com/umhprqe (accessed on 15 March 2026).

Microaxial flow pumps (e.g., Impella) are percutaneous MCS devices used in CS to assist left ventricular function by unloading the heart and improving CO [73]. It consists of a catheter-mounted axial flow pump that draws blood from the left ventricle and ejects it into the ascending aorta, reducing myocardial workload and oxygen demand while improving blood flow and enhancing systemic organ perfusion (Figure 4) [73,74]. These devices are commonly used in AMI-CS, cardiomyopathy with acute decompensation, postcardiotomy CS and during high-risk PCI to stabilise the patient’s haemodynamic state. By decreasing ventricular pressure and wall stress, it promotes myocardial recovery or serves as an intermediate to definitive therapies.

Contemporary microaxial flow pump support encompasses devices with differing haemodynamic capabilities and clinical indications. Impella CP, typically deployed percutaneously, provides a flow rate of up to ~4.0 L/min [73] and short-term left ventricular unloading and is most commonly used in acute CS for rapid stabilisation. In contrast, Impella 5.5 delivers higher flow rates (up to ~5.5 L/min) and is often surgically implanted or placed via axillary access enabling more durable haemodynamic support and greater left ventricular unloading [75,76].

This distinction is particularly relevant in CS due to mechanical complications, such as VSD, where more sustained and robust unloading may be required as a bridge to definitive surgical repair. In such scenarios, Impella 5.5 may be preferred over Impella CP due to its higher flow capacity and suitability for prolonged support, whereas Impella CP may be limited in the presence of large left-to-right shunts or profound haemodynamic compromise. Device selection should therefore be individualised based on shock severity and risk of complications, anticipated duration of support, and underlying aetiology.

Recent studies underscore the importance of careful patient selection, as Impella devices have been associated with substantial risks of adverse events and high costs [77,78,79].

The DanGer-SHOCK trial, published in The New England Journal of Medicine, was an international, open-label RCT evaluating the effectiveness of early mechanical circulatory support with the Impella CP device in AMI-CS patients [80]. The trial enrolled 360 patients (final analysis included 355 patients; 179 in the Impella CP group and 176 in the standard-care alone group) and compared early use of a microaxial flow pump (Impella CP) run at the highest possible performance for greater than or equal to 48 h alongside standard care against standard care alone, which included inotropes, vasopressors, MCS and revascularisation as needed.

The primary endpoint was death from any cause at 180 days and the study showed a significant difference in all-cause mortality between the two groups (Impella 45.8% vs. medical therapy 58.5%, *p* = 0.04) [80]. However, major bleeding, limb ischemia, haemolysis, device failure and worsening aortic regurgitation were more common in the Impella CP group compared to standard care alone. Furthermore, in the recent long-term (10 year) follow up presented at the ESC Congress 2025, the results showed durable survival benefit with routine Impella CP in STEMI-CS when compared to standard care alone with an absolute mortality reduction of 16.3% (HR ≈ 0.70) [81]. Overall, the findings suggested that the routine early use of Impella CP in AMI-CS improves survival but device-related complications remain relevant, and therefore careful patient selection and experienced centres are key. Similarly, the EuroSHOCK Registry is a multicentre, observational study looking at 120 patients from 14 tertiary centres across five European countries, which demonstrated further evidence in support of the use of Impella in AMI-CS, showing a reduction in lactate levels suggestive of improvements in organ perfusion [82]. Impella devices may therefore produce meaningful improvements in haemodynamics in AMI-CS that translate into improved mortality, however at the cost of increased complications compared to standard care. Further RCTs are needed to confirm the efficacy of Impella devices and better understand optimal patient selection.

##### MCS Device Comparison and Current Evidence

Despite various studies supporting the use of MCS in the acute stage of CS management, there is currently little evidence to support the exclusive use of one type of MCS over another (Table 1). MCS use varies significantly between countries; for example, while there is an increasing use of ECMO in France, the NCSI registry above reports that Impella makes up the majority of MCS in the US [16]. There are also currently ongoing trials such as the “Assessment of ECMO in acute myocardial infarction with Non-reversible Cardiogenic shock to Halt Organ failure and Reduce mortality” (ANCHOR) trial looking at the combined use of both ECMO and IABP in AMI-CS to improve mortality rates [83] as previously suggested by other studies [84,85]. International differences and the lack of data highlight the need for further research to optimise the use of MCS in CS as this may be the key to improving survival rates.

#### 3.2.3. Revascularisation

In the context of AMI-CS, PCI and coronary artery bypass grafting (CABG) are two revascularisation procedures that are considered with the aim of restoring myocardial perfusion, improving CO, and preventing further ischemic damage. The choice between PCI and CABG depends on the patient’s clinical stability, coronary anatomy, and institutional resources. The revascularisation strategies discussed in this section apply specifically to AMI-CS and are not relevant to non-ischaemic causes of CS in which management priorities differ.

##### Percutaneous Coronary Intervention (PCI)

PCI is the first-line revascularisation strategy for AMI-CS patients [3]. The procedure quickly restores blood flow to the ischemic myocardium, which is critical in minimising myocardial damage and improving survival in the setting of haemodynamic instability [3,86]. By re-establishing coronary blood flow, PCI reduces myocardial ischemia, enhances cardiac contractility, and improves CO, alleviating the cycle of hypoperfusion. PCI can be performed rapidly in an emergency, making it the preferred option in the acute phase of CS when time is critical to save viable myocardium.

In CS, the guidelines recommend PCI targeting the culprit lesion (the most severely blocked artery) responsible for the ischemia. This follows evidence from the CULPRIT-SHOCK trial, which compared immediate multi-vessel PCI against culprit-lesion-only PCI in 706 multi-vessel CAD patients presenting with AMI-CS [6]. The trial demonstrated better outcomes when PCI was limited to the culprit lesion rather than addressing multiple lesions during the acute phase at 30-day mortality (45.9% vs. 55.4%, *p* = 0.03) as well as at 1-year follow up (52.0% vs. 59.5%) [87]. Following these results, immediate multi-vessel PCI should be avoided due to increased risk of adverse outcomes and non-culprit lesions can be addressed in a staged procedure following patient stabilisation.

Despite its benefits, PCI alone may not fully restore cardiac function in patients with extensive myocardial damage or multi-vessel disease [6]. In these cases, additional therapies such as the MCS devices mentioned above (e.g., ECMO or Impella) may be used concurrently to aid in stabilising the patient perioperatively and post-procedure.

Antiplatelets

Antiplatelet therapy is a fundamental component of AMI, forming the cornerstone of dual antiplatelet therapy (DAPT) to prevent stent thrombosis and recurrent ischaemic events following PCI. In AMI-CS, robust evidence to guide optimal AMI-CS has been lacking, with current practice largely taken from non-shock AMI populations. Gastrointestinal hypoperfusion, delayed absorption, vomiting, and altered drug metabolism commonly seen in shock can result in unpredictable and delayed platelet inhibition, increasing the risk of early thrombotic complications.

The recent DAPT-SHOCK-AMI trial, presented at ESC Congress 2025, was the first randomised study on antiplatelet therapies in AMI-CS patients undergoing PCI. The multicentre, double-blind study sought to determine whether rapid, reliable platelet inhibition with IV cangrelor could improve early outcomes compared with standard crushed ticagrelor due to its quicker onset and action independent of gastrointestinal absorption. IV cangrelor demonstrated significantly greater and faster platelet inhibition and early procedural observations suggested trends toward improved coronary flow and fewer thrombotic events during PCI. Rates of major bleeding were comparable, supporting the safety and feasibility of intravenous cangrelor in this critically ill cohort. However, no statistically significant difference was observed in the primary composite endpoint of death, MI, or stroke at 30 days. These findings provide important insight into optimising antiplatelet therapy in AMI-CS, a population in whom delayed oral absorption may undermine conventional treatment. IV cangrelor represents a rational bridging strategy until patients stabilise and can transition to oral agents.

##### Coronary Artery Bypass Grafting (CABG)

CABG, involves surgically creating new pathways for blood to bypass blocked or stenosed coronary arteries, improving perfusion to ischemic myocardial tissue [88]. CABG is generally used for patients with severe multi-vessel disease or left main coronary artery stenosis, which cannot be adequately treated with PCI [3,43,89].

Healthy blood vessels, typically the internal mammary artery or saphenous vein, are grafted to bypass the blocked arteries, ensuring a stable and long-term blood supply to the myocardium [88]. By restoring perfusion to a larger area of ischemic myocardium, CABG can improve myocardial contractility and reduce the progression of left ventricular pump failure.

CABG is often less suitable in the acute phase of CS because the procedure is time-intensive, delaying revascularisation; the risks of perioperative mortality are higher in patients with profound haemodynamic instability or multi-organ failure; and cardiopulmonary bypass, commonly used during CABG, can exacerbate systemic inflammation and organ dysfunction in unstable patients [89,90]. CABG is typically performed in patients who survive the initial phase of CS and are stabilised with pharmacological or mechanical support. CABG is also considered in cases where PCI has failed or is not feasible, such as in patients with complex coronary anatomy.

Early and aggressive revascularisation remains central to improving outcomes in AMI-CS. Several key RCTs and registries have demonstrated that rapid culprit-lesion PCI or CABG confer significant survival advantages in CS. The SHOCK trial looked at evaluating the impact of early revascularisation (PCI or CABG) versus initial medical stabilisation in AMI-CS cases [4]. Early revascularisation significantly reduced 6-month mortality compared to medical therapy alone (50.3% vs. 63.1%, *p* = 0.027). Furthermore, at 1 year, the survival benefit persisted, with a 13.2% absolute reduction in mortality (46.7% vs. 33.6%, *p* = 0.03). These findings have been consistently supported by registry data and form the basis of current guideline recommendations, which emphasise early revascularisation as a Class I indication in AMI-CS. Together, these data highlight that timely mechanical reperfusion remains the cornerstone of CS management.

## 4. Patient Outcomes

### 4.1. Mortality

CS remains one of the most critical and challenging acute conditions in cardiology to manage. The prognosis for patients who experience CS is influenced by several factors, including the underlying cause of the shock, the timeliness and type of treatment received, and the patient’s overall health status. Survival rates, recurrence risk, and morbidity outcomes can vary based on treatment strategies as well as other clinical and demographic factors (Table 2).

The acute phase of CS is associated with high mortality, and early intervention is crucial to improving survival outcomes. The ESC-HF-LT Registry, a prospective, observational study, included 6629 HF patients, of whom 2.9% presented with CS [91]. Among these patients, CS had the highest in-hospital all-cause mortality rate at 36.1%. Additionally, nearly half (49%) of all in-hospital deaths in CS patients occurred within the first 24 h of presentation, highlighting the high mortality at presentation and the rapidly progressive nature of the condition.

The ESC-HF-LT Registry also indicated a notably high 1-year mortality rate for CS patients at 54.0%, underscoring the severe prognosis associated with this clinical profile despite acute management [91]. These findings emphasise the need for early and aggressive intervention in CS patients to improve outcomes and long-term survival.

Internationally, the National Cardiogenic Shock Initiative (NCSI), a multicentre registry based in the United States, reported a significantly lower in-hospital mortality (28% vs. historical rates of 40–50%) where patients received early MCS, predominantly Impella, and revascularisation [92]. The study revealed various best practices for AMI-CS which align with the outcomes of the SHOCK trial, e.g., early diagnosis and rapid stabilisation of systemic perfusion with vasopressors and inotropes, MCS Pre-PCI and more protocolised, evidence-based care.

**Table 2:** Key registry and trial data on cardiogenic shock mortality.

Registry/Trial	Years	N	Aetiology (%)	Mortality (%)	Follow Up (%)	Notes
SHOCK trial [4]	1999	302	AMI-CS	30-day: 50.3 (early revascularisation) vs. 63.7 (medical stabilisation)	1 y: 33.6 vs. 46.7	Early revascularisation improved survival
CCCTN (HF-CS registry) [27]	2017–2023	8974	HF-CS: 59, AMI-CS: 27	-	-	Demonstrates aetiological shift towards HF-CS dominance
IABP-SHOCK II [5]	2012	600	AMI-CS	30-day: 39.7 (IABP) vs. 41.3 (control)	1 y: 52 (IABP) vs. 51 (control)	No benefit with IABP
DanGer-SHOCK [80]	2024	355	AMI-CS	180-day: 45.8 (Impella CP) vs. 58.5 (standard care)	10 y: 16.3 mortality risk reduction	Impella showed survival benefit
NCSI (US) registry [92]	2016–2020	406	AMI-CS	In-hospital: 28	30-day: 32; 1 y: 47	Early MCS + PCI protocols lowered mortality
CULPRIT-SHOCK [6]	2017	706	multi-vessel CAD AMI-CS	30-day: 45.9 (culprit-only) vs. 55.4 (multi-vessel)	1 y: 52.0 (culprit-only) vs. 59.5 (multi-vessel)	Culprit-only PCI superior to multi-vessel

Large epidemiological studies demonstrate marked temporal shifts in the incidence and in-hospital outcomes of CS over the past two decades. Analysis of the U.S. National Inpatient Sample between 2004 and 2018 identified more than 1.2 million CS hospitalisations, representing approximately 0.2% of all admissions, and showed that the incidence of CS hospitalisation more than tripled during this period from 122 per 100,000 hospitalisations in 2004 to 408 per 100,000 in 2018 (*P*_trend_ < 0.001) [93]. Importantly, this increase was accompanied by a gradual but consistent decline in adjusted in-hospital mortality, falling from approximately 49% to 37% overall. Mortality reductions were observed in both AMI-related and non-AMI CS, and were consistent across demographic groups, hospital characteristics, and geographical regions. These data reflect both an increasing recognition and coding of CS, as well as incremental improvements in acute management strategies and systems of care over time. However, despite these gains, CS continues to carry a substantial absolute mortality burden, underscoring the need for ongoing improvements in early diagnosis, coordinated management pathways, and advanced supportive strategies.

### 4.2. Long-Term Quality of Life

The quality of life in patients who survive CS is often significantly diminished with ongoing physical, psychological and social challenges. There is extensive data on mortality in AMI-CS; however, there is little research looking into the long-term quality of life impact of CS.

A retrospective cohort study was conducted in Ontario, Canada, examining the long-term outcomes of 9789 AMI-CS patients who were admitted to the ICU [94]. The study demonstrated the high long-term morbidity following discharge with 42% of patients needing a higher level of care compared to baseline as well as a readmission rate of 47.5% within 1 year. This could be a consequence of prolonged HF or the effects of MCS and resuscitation measures, highlighting the need for improvements in medical intervention to reduce morbidity following discharge.

The Circulation article titled “Survivorship After Cardiogenic Shock”, described the unique nature of impairment following CS, specifically Post-Intensive Care Syndrome (PICS) [95]. PICS consists of the cognitive, physical and mental impairment that follow recovery from critical illness and how it can significantly impact a patient’s quality of life. In CS patients, the combination of intensive MCS use, recurrence of MI or cardiac arrest as well as the fear and anxiety associated with those re-occurring can have a significant psychological impact on a patient.

## 5. Burden on Healthcare Providers

CS management places a substantial burden on healthcare providers, resulting in significant financial costs and extensive healthcare resource utilisation. The management of CS often requires prolonged ICU stays, specialised personnel and equipment, and advanced interventions such as MCS, all of which contribute to its high cost. Here we provide an illustration of current costs within the National Health Service (NHS), the publicly funded health system in the UK.

### 5.1. Financial Costs of Care

The financial cost of CS in the UK is not well-documented in primary research, particularly in terms of national data. While international studies provide insights into the costs associated with CS, there is a lack of similar comprehensive studies within the UK context. For instance, the retrospective population-based cohort study conducted in Ontario also evaluated the healthcare costs and resource utilisation of AMI-CS between 2009 and 2019 [96]. The study reported a median inpatient cost of £17,562 ($23,912) per AMI-CS patient, with total 1-year inpatient costs reaching up to £48,901 ($66,582) for survivors to discharge. However, such detailed cost analyses are scarce in the UK, highlighting a significant gap in the understanding of the economic burden of CS on the NHS.

The acute management of CS typically involves prolonged stays in ICUs, driven by the need for invasive haemodynamic monitoring, vasoactive medications, MCS and continuous multidisciplinary care (Table 3). The average daily cost of an ICU bed in the NHS is estimated to be approximately £1881; however, this varies depending on patient complexity and required interventions [97]. Given that CS patients often remain in ICU for extended periods, these costs accumulate rapidly, contributing significantly to the overall financial burden.

MCS devices are frequently used in CS to stabilise patients or serve as a bridge to recovery or further intervention; however, these technologies are costly both in terms of initial cost and ongoing maintenance or utilisation. For example, the implantation of a VAD in the UK costs approximately £63,830, with additional monthly maintenance costs of around £1069 [98]. ECMO is also an expensive treatment method requiring highly specialised staff and equipment, further elevating system-wide costs. In Ontario, Canada, public funding for ECMO in CS patients over five years was projected to cost approximately £1.23 million ($1.67 million) for treating 314 individuals [99]. Available evidence on ECMO costs in the UK is limited; one modelling study reported per-patient costs of £8616–£28,829 (2012 prices) for adult HF, depending on the device and indication, but did not account for several major cost components, including staffing, complications and long-term care [100]. Consequently, current ECMO costs are likely to be substantially higher. Likewise, publicly available UK cost data for contemporary microaxial flow pumps such as Impella CP and Impella 5.5 (which now supersede the Impella 2.5) are limited; NICE previously reported the cost of a single-use Impella 2.5 as approximately £15,000 while the reusable Automated Impella Controller, which is needed to use the device, costs approximately £35,000 [101].

Beyond the direct procurement costs, the reimbursement landscape for these advanced therapies adds a layer of complexity to their provision. Currently, the NHS nationally commissions ECMO services specifically for severe respiratory failure [102]; however, ECMO for HF is not a nationally commissioned service [103]. Consequently, cardiac ECMO is generally delivered within centres already commissioned for respiratory support and likely relies on centre-specific funding arrangements rather than a centralised national stream. Similarly, Impella devices are not nationally commissioned, making their availability dependent on individual trust-level funding and business cases. This reliance on local rather than national funding models possibly contributes to the regional disparities in access to advanced MCS.

The pharmacological management of CS includes the use of vasoactive agents (e.g., NE, dopamine), anticoagulants, and supportive medications. While specific, current NHS-wide cost data for these drugs are limited, and the combination of high doses and prolonged administration in the ICU setting contributes substantially to the overall expenditure, particularly when compounded by the duration of critical care stays.

### 5.2. Resource Utilisation

The demand for ICU beds by CS patients is considerable, often displacing other critical cases. With an estimated 6110 critical care beds in England, approximately 66% of which are adult beds, capacity is often stretched, especially in urban centres or during winter pressures [104].

The effective management of CS requires input from interventional cardiologists, intensivists, and HF specialists. With increasing CS incidence driven by ageing populations and rising rates of CAD, there is growing pressure on an already strained cardiology workforce. The British Heart Foundation conducted the first census of the cardiac workforce in England highlighting shortages in consultant cardiologists, further raising concerns about the system’s ability to meet demand in the context of CS [105].

The care of CS patients requires access to advanced technologies such as echocardiography, catheterisation labs, and MCS devices. However, the availability of such equipment varies across NHS trusts, with some hospitals, especially in rural areas, lacking access to life-saving interventions like ECMO or advanced VADs. This contributes to geographic disparities in care provision.

### 5.3. Regional Disparities

Access to critical care and cardiology services vary significantly by region. Larger, specialised centres in urban areas often have better access to advanced technologies and experienced personnel, whereas smaller or rural hospitals may be under-resourced. These disparities can delay diagnosis and treatment, adversely impacting patient outcomes. Variations in the number of ICU beds and the distribution of cardiology and cardiac surgery departments further exacerbate these challenges. Furthermore, the 2021 GIRFT cardiology report highlighted delays in HF management and regional variability in access to advanced cardiac services, which may worsen CS outcomes in under-resourced trusts [106].

## 6. Current Strategies for Prevention

Preventing CS remains a challenge, given its often sudden and acute onset. However, strategies in cardiovascular disease prevention and HF management form the cornerstone of reducing its incidence.

A major preventive focus is the optimisation of chronic HF management, particularly in high-risk individuals with reduced ejection fraction. Adherence to guideline-directed medical therapy (GDMT) using ACE inhibitors, beta-blockers, SGLT2 inhibitors, and mineralocorticoid receptor antagonists (MRAs) has demonstrated significant reductions in mortality and rehospitalisations in landmark trials [107,108,109,110,111]. In the UK specifically, the NHS Heart Failure Pathway promotes early identification, primary care optimisation, and timely referral to HF clinics [112].

Additionally, the management of acute coronary syndromes (ACS), the most common underlying cause of CS, can mitigate progression to shock. Early PCI [113], use of high-sensitivity troponin assays [114], and rapid ambulance-to-cath lab protocols (as employed by the London Chest Pain Pathway) [115] are key public health strategies.

Public education on early symptom recognition (e.g., chest pain, breathlessness), improved access to emergency care, and enhanced uptake of cardiac rehabilitation programmes can also play preventive roles [116,117]. These services reduce delays in treatment, promote swift recovery and reduce the risk of recurrent cardiovascular events.

Risk factor control at a population level, such as aggressive management of hypertension, diabetes [118], smoking cessation [119] and lipid lowering therapy (NICE Statin recommendation) [120], is essential to reduce the burden of CAD, which predisposes to CS. Initiatives such as NHS England’s Cardiovascular Disease Prevention programme [121] also aim to identify and manage these modifiable risks through initiatives like the NHS Health Check [122,123].

While not all CS is preventable, especially in cases of acute valvular disease or myocarditis, these strategies contribute significantly to reducing the incidence and severity of cases that progress to CS.

## 7. Future Directions

Improvements in trial design and early diagnostic techniques for CS are crucial for reducing mortality and optimising treatment outcomes. Early identification of CS can facilitate timely interventions, prevent complications, and reduce the subsequent burden on healthcare resources. Several strategies and advancements in diagnostic techniques are being explored and implemented as outlined below.

### 7.1. Improving Trial Design

Many recent RCTs have failed to demonstrate clear survival benefits reflecting the heterogeneity of CS and inconsistencies in study design. Recent consensus from the sixth Critical Care Clinical Trialists (3CT) Workshop outlined several key strategies to improve trial design, making studies more standardised, precise and patient-centred and subsequently strengthening the evidence base in CS management [124].

A central recommendation is to streamline CS classification and severity scoring to allow for a standardised framework for treatment, enrolment and outcome measurement. The SCAI staging system serves as a reproducible and dynamic framework for identifying shock stage progression and its universal implementation could provide consistency in patient stratification.

The 3CT also highlighted phenotyping and endotyping as an approach to enrich inclusion criteria where identifying patients with specific phenotypes or biomarkers such as interleukin-6 (IL-6) and dipeptidyl peptidase-3 (DPP-3) could allow for the creation of distinct subgroups alongside their benefit of representing severity and prognosis as well as providing therapeutic targets.

Another key message was the expansion and diversification of endpoints. While short-term mortality has traditionally been the primary endpoint of most RCTs, it fails to represent the full clinical impact of interventions despite being objective and measurable. Endpoints should be optimised to include broader, composite endpoints that include measures such as device-free survival, neurological recovery, and patient-reported outcomes (PROs) to reflect meaningful, holistic, long-term benefit.

Finally, innovative statistical and operational designs can enhance both feasibility and interpretability. The use of Bayesian approaches, win-ratio analyses, and adaptive or registry-embedded designs can improve efficiency, manage small sample sizes, and generate results that are both clinically meaningful and generalisable. Embedding trials within existing registries or clinical networks would streamline enrolment, lower costs, and ensure applicability to real-world practice.

### 7.2. Biomarker-Driven Subphenotypes

Traditional management strategies often take a “one-size-fits-all” approach; however, growing evidence suggests that patients with CS present with diverse pathophysiological mechanisms, treatment responses, and outcomes as represented by recent efforts with the SCAI shock stages [7]. As previously mentioned, the current CS clinical definition is yet to be standardised across clinical practice and has inspired an interest in biomarker-driven subphenotyping as a strategy to enable more precision management of CS patients.

Recent studies have demonstrated that biological subphenotypes in CS, identified through clustering of clinical and biochemical markers, can provide meaningful distinctions in terms of disease severity and prognosis. For example, a 2024 study published in *The Lancet* used unsupervised machine learning on biomarker profiles in patients with CS and identified distinct subgroups with significantly different 90-day mortality rates [125]. The researchers identified four biomarker-driven CS subphenotypes (“adaptive”, “non-inflammatory”, “cardiopathic”, and “inflammatory”), each associated with varying 28-day mortality rates. These subphenotypes were characterised by varying degrees of inflammation, organ dysfunction, and myocardial stress. Notably, the “inflammatory” and “cardiopathic” subphenotypes exhibited the highest mortality. The study highlighted that incorporating subphenotype classification into traditional risk models, such as the SCAI shock stages, improved risk stratification, suggesting that classifications may eventually guide individualised therapeutic decisions, helping clinicians better identify patients more likely to benefit from aggressive MCS or targeted pharmacological therapies.

The identification of these subphenotypes also has implications for clinical trial design. Subphenotyping offers the opportunity to stratify patients more effectively and improve trial precision, potentially increasing the likelihood of demonstrating benefit in targeted populations. A study by Zweck et al. (2023) examined the clinical course of patients in CS stratified by machine learning-derived phenotypes [126]. The findings demonstrated that the phenotypes identified through supervised machine learning are universally applicable and could guide the development of future clinical trials supporting the creation of management algorithms tailored to each specific CS phenotype.

Furthermore, dynamic biomarker monitoring may allow clinicians to track patient trajectories and assess responses to therapy in real time [127]. Serial measurements of arterial lactate, for example, may help determine whether a patient is transitioning between phenotypes or responding to a given intervention. This could further refine treatment escalation or de-escalation strategies, such as when to initiate, wean, or withdraw MCS.

While biomarker-driven subphenotyping in CS is still in its early stages, its promise is significant and could make CS management more personalised and dynamic. Moving forward, large-scale validation studies will be necessary to confirm the clinical utility and safety of applying a subphenotype-specific approach.

### 7.3. AI and Predictive Analytics

AI and predictive analytics are emerging as transformative tools in the diagnosis, risk stratification, and management of CS. Given the high mortality associated with CS and the critical need for timely interventions, AI-driven models have the potential to greatly enhance clinical decision-making by identifying patients at risk earlier and optimising treatment pathways based on individualised data.

One of the key applications of AI in CS is early prediction. Traditional risk scores, such as the IABP-SHOCK II or SCAI staging system, are valuable but limited by static variables and subjective interpretation. Machine learning (ML) algorithms, however, as used in the development of biomarker subphenotypes, can process vast amounts of clinical data—including vital signs, laboratory values, and imaging findings—to detect subtle patterns that precede clinical deterioration. For instance, the STOPSHOCK score, developed using ML techniques, is a continuously updated, AI-based scoring system which has demonstrated high accuracy in predicting the development of CS in hospitalised ACS patients [128,129]. As of early 2025, the STOPSHOCK 2.0 model is currently undergoing external validation, with over 6500 patients enrolled in the study. Such tools enable earlier recognition and escalation of care, potentially before overt clinical signs of shock manifest.

AI is also being incorporated into echocardiography which is an imaging modality used extensively in CS. Echocardiography enhanced with AI algorithms can automatically quantify left ventricular function as well as detect regional wall motion abnormalities, as suggested in several validation studies [130], which may prove useful in evaluating the aetiology and severity of CS. This reduces dependence on expert interpretation and ensures timely, accurate assessments even in resource-limited settings.

However, the adoption of AI in CS is not without challenges. Issues related to suboptimal image quality, model interpretability, and integration with clinical workflows remain significant barriers. Additionally, AI models are trained on existing retrospective datasets from high-resource centres, which may limit their generalizability. Prospective validation and clinician acceptance are essential before these tools can be widely implemented in routine practice.

### 7.4. Smart Wearable Devices

Wearables are gaining ground in cardiovascular care and may hold future applications in early detection and may prove to be revolutionary in the outpatient monitoring of patients at risk of developing CS. Devices such as the Apple Watch [131], KardiaMobile [132] and the Zio patch [133] offer continuous ECG monitoring and can detect arrhythmias, HR variability, and signs of decompensation. In patients with chronic heart failure—who are at increased risk of progressing to CS—these wearables could alert patients and providers to early warning signs like sustained tachycardia or reduced HR variability.

One particularly promising innovation is the development of implantable haemodynamic monitors, such as the CardioMEMS device. This device is implanted within the distal pulmonary artery via right heart catheterisation and will remotely monitor pulmonary artery pressures [134]. While currently approved for patients with chronic heart failure to detect worsening haemodynamics, such devices may eventually be adapted for use in CS or post-CS recovery, allowing for early detection of haemodynamic deterioration and preventing rehospitalisation.

Wearable and smart technologies also empower patient self-monitoring and engagement [135], a critical component in long-term recovery after an episode of CS. Mobile apps that sync or are used in conjunction with smart devices can track symptoms, medication adherence, and physiological trends, transmitting data to clinicians and enabling early intervention when needed [136].

Despite their promise and high sensitivity, these technologies face several limitations and have been questioned on their false positive rates. For example, in smart wearable devices for the detection of atrial fibrillation, device inaccuracies and false alerts may lead to increased use of resources via testing and subsequent increased healthcare costs [137,138]. Similarly, false-negative readings in symptomatic patients may delay intervention and necessitate more invasive or costly procedures. In addition, most wearables are currently validated only in stable ambulatory populations, and their role in critically ill patients remains largely experimental. Furthermore, integrating the vast volume of data generated into meaningful clinical insights requires robust infrastructure, clinician training, and rigorous validation.

### 7.5. Shock Teams and Standardised Pathways

Structured, multidisciplinary shock teams have emerged as an effective model to improve patient outcomes in CS by enabling rapid decision-making, protocolised escalation, and coordinated resource use by bringing multiple designated providers together simultaneously via one call to reduce delays in care and allowed for shared expertise [139]. The recommended core members of the CS team would include interventional cardiologists, cardiac intensivists, advanced HF specialists and cardiothoracic surgeons alongside respiratory therapists, echocardiographers, perfusion/ECMO specialists, emergency medicine, palliative care consultants as well as medical and surgical specialties.

Importantly, observational evidence demonstrates that shock team implementation is associated with improved survival outcomes. In 250 patients with refractory CS treated with short-term MCS, implementation of a multidisciplinary shock team was associated with a significant improvement in short-term survival (3-month survival rate: 63% vs. 54%) and long-term survival (1-year survival rate: 59% vs. 45%, *p* = 0.043), with lower mortality observed at follow up compared with the historical cohort [140]. In this cohort, structured shock team care was also associated with more consistent device selection and earlier escalation of support where the time required to perform implantation of MCS, following shock team introduction, decreased by more than 3 h. Additional multicentre observational studies have also reported that centres with established shock teams experience lower in-hospital mortality, overall less use of MCS and increased initiation of advanced MCS (e.g., ECMO and Impella) compared with centres without formalised shock team models [141].

These principles have been highlighted within national frameworks such as the Shock to Survival report in the UK, which outlines a structured, systems-based approach to CS care [44]. This framework emphasises early recognition, standardised classification, rapid access to multidisciplinary expertise and intervention, and regionalised network models of care mirroring successful NHS models such as heart attack centres (HACs), trauma networks, and stroke services. The framework is built around the principle of delivering the right intervention to the right patient at the right time based on the right data and through the right specialists via coordinated, networked care rather than isolated hospital-based management. Following recognition of possible CS at non-specialist hospitals or HACs, via elevated National Early Warning Score (NEWS) 2 scores (≥5) with evidence of hypoperfusion, the report recommends early ECG and focused cardiac ultrasound (FoCUS) to rapidly identify cardiac dysfunction and reversible causes, enabling timely escalation and preventing progression to multi-organ failure. Disease severity should be classified using the SCAI shock staging to support consistent triage, inter-hospital transfer and timing of advanced therapies. CS multidisciplinary teams (CS-MDTs) are a central component of the framework and are based at CS centres, where they provide rapid, continuous, 24/7, multidisciplinary input to support specialist decision-making. These teams typically include an interventional cardiologist, a cardiac intensivist, cardiac critical care nursing staff, a HF cardiologist and a cardiac surgeon as well as, where appropriate, a member of the regional transplant team or specialist palliative care, all working together to optimise individual patient CS management. The report advocates regional network models of CS care, recognising that no single hospital can safely deliver the full spectrum of CS management. Within these networks, patients identified and triaged at non-specialist hospitals or HACs are escalated to designated CS centres, with onward referral to advanced HF centres (AHFCs) when required for advanced therapies such as durable MCS or transplantation. Effective CS networks require structured design, governance, and leadership, supported by clear referral triggers, agreed escalation pathways and robust transfer and retrieval systems. The report recommends building on the existing infrastructure, alongside designated CS centre leads, workforce training, and expansion of FoCUS capability to ensure sustainability, while repatriation to local hospitals once specialist care is no longer required supports capacity and continuity of care.

The systems-based approach outlined in the Shock to Survival report aligns closely with broader UK consensus guidance addressing other time-critical cardiac emergencies in which CS is a common complicating feature. The British Cardiovascular Intervention Society Consensus for out-of-hospital cardiac arrest (OHCA) emphasises the role of designated cardiac arrest centres (CACs), early identification of shock states including SCAI staging, and structured pathways enabling rapid access to coronary intervention, critical care, and MCS where appropriate [142]. Similarly, the Joint British Societies’ recommendations for cardiac arrest and emergencies in the cardiac catheterisation laboratory explicitly highlight CS as a key determinant of prognosis and advocate early multidisciplinary decision-making, escalation to specialist centres, and coordinated post-resuscitation care [143]. More recently, UK multi-society consensus statements on left-sided Impella support and on the management of emergencies in patients receiving ECMO further reinforce the principles of early recognition, prompt escalation for multidisciplinary support, team prioritisation and rapid, coordinated intervention [144,145]. Collectively, these UK-specific recommendations support a unified model of care in which CS, whether occurring in isolation or in association with cardiac arrest, is managed through regionalised specialist networks rather than isolated institutional practice.

Recent proposals, including the Cardiogenic Shock Team Collaborative, advocate for regional networks that align care pathways, share performance metrics, and promote continuous learning across centres [139]. Parallel to this, early experience from established CS programmes in the US shows that consistent activation criteria, defined leadership, and data-driven feedback loops are key for successful care improvement [146].

Future shock teams should evolve towards integrating shared protocols and standards so that each centre works homogenously, e.g., by applying similar escalation, de-escalation and device-weaning algorithms. Recommendations also include performance benchmarking with quality indicators such as time to shock team activation, time to MCS and adherence to protocol as a means of identifying and addressing outliers in optimal care. Embedding prospective data collection within these networks would facilitate pragmatic trials and enable iterative refinement of care. As with trauma and cardiac arrest systems, formalising shock teams at institutional and regional levels represents a critical next step toward achieving timely, evidence-based, and reproducible patient outcomes in CS (Table 4).

## 8. Conclusions

This review highlights the complexity and importance of CS, a condition that, despite medical advances, continues to be associated with high morbidity and mortality. An analysis of current clinical practices reveals that while early intervention strategies, such as prompt revascularisation and pharmacological support, improve outcomes, survival rates remain suboptimal. The evidence surrounding MCS devices remains mixed, with a need for more well designed RCTs.

Beyond clinical care, this review demonstrates the burden that CS places on the healthcare providers, providing an overview of this issue within the UK NHS. Significant regional disparities in access to advanced interventions such as ECMO compound these challenges. Emerging technologies, including biomarker-driven subphenotyping, AI, and wearable devices, show promise for earlier detection and more personalised management; however, their adoption into routine practice remains in its infancy.

Going forward, improvements in CS outcomes will likely depend on a multifaceted approach: the development of national CS networks to reduce inequities and delays in care, investment in preventative public health strategies targeting cardiovascular disease (CVD), and rigorous integration of technological innovations into standard care pathways. Future research should prioritise precision-based management strategies and explore the long-term quality of life in survivors of CS, which remains a poorly understood area. Ultimately, bridging the gap between innovation, clinical practice, and health system infrastructure offers the best hope for improving outcomes for patients.

## Figures and Tables

**Table 1:** Comparison of key mechanical circulatory support (MCS) device trials.

MCS Device	Mechanism	Key Trials	Shock Aetiology	Key Inclusion/Exclusion Criteria	Main Findings	Key Limitations
IABP	Balloon counterpulsation; aortic inflation during diastole, deflation in systole	IABP-SHOCK II	AMI-CS	AMI with CS undergoing early revascularisation	No mortality benefit at 30 days or in long term	Limited haemodynamic support; not effective in severe CS
ECMO	Extracorporeal circulation; blood drained, pumped through membrane oxygenator via a centrifugal pump, then returned	ECLS-SHOCK	Predominantly AMI-CS	Profound CS with high lactate; excluded prolonged cardiac arrest	No mortality benefit vs. standard care	Increased bleeding, peripheral ischemia
Impella CP	Catheter-mounted axial flow pump; draws blood from LV, ejects into ascending aorta	DanGer-SHOCK	STEMI-CS	STEMI, reduced LVEF, early presentation; excluded severe neurological injury	Reduced 180-day mortality vs. standard care	Narrow population; high bleeding and vascular complications (major bleeding, limb ischemia, haemolysis, device failure)

**Table 3:** Estimated economic costs of cardiogenic shock management.

Resource	Estimated Cost (UK)	Notes
ICU bed/day	£1881	Higher for advanced care and prolonged stays
Impella 2.5	£15,000 + £35,000 controllers	NICE cost estimate for single use and console; Impella 2.5 has been superseded by Impella CP and 5.5, which likely incur equal or higher costs (costs not freely published but estimated £20,000–£40,000+)
ECMO	£8616–£28,829+ (2012 prices)	Device-related modelling estimate only; excludes staffing, ICU stay, complications; contemporary costs likely substantially higher
Ventricular assist device (VAD) implantation	£63,830 + £1069/month	Long-term maintenance costs significant
Vasoactive agents (e.g., noradrenaline, dobutamine, milrinone)	Variable—typically hundreds to low thousands of £ per patient	Costs accumulate with prolonged ICU use and high dosing; compounded by critical care stay duration

**Table 4:** Summary of key management approaches in cardiogenic shock and associated outcomes.

Key Components	Main Evidence/Trials	Impact on Outcomes	Clinical Considerations
**Early diagnosis & risk stratification**			
Rapid clinical assessment, haemodynamic evaluation, SCAI staging, biomarkers, echocardiography	SCAI classification studies	Earlier recognition is consistently associated with improved survival; dynamic staging during first 24 h enhances prognostic accuracy	Serial reassessment is critical; early staging helps guide escalation decisions and timely intervention
**Pharmacological therapy**			
Vasopressors (e.g., noradrenaline), inotropes (dobutamine, milrinone, levosimendan)	DOREMI	Stabilises perfusion and buys time but no proven mortality benefit as stand-alone therapy	Best used as bridge to revascularisation or MCS; prolonged use increases arrhythmia, ischemia, and metabolic complications
**Revascularisation**			
Culprit-lesion PCI; CABG when indicated	SHOCK trial; CULPRIT-SHOCK	Early PCI reduces mortality at 30 days and 1 year; rapid reperfusion interrupts the downward spiral of shock	Culprit-only PCI is preferred acutely; CABG reserved for selected patients after stabilisation or failed PCI
**Mechanical circulatory support (MCS)**			
IABP, Impella, ECMO, combinations	IABP-SHOCK II; DanGer-SHOCK; ECLS-SHOCK	Impella CP improves survival in AMI-CS; ECMO restores perfusion but without clear survival benefit	Device choice depends on patient profile, timing, and centre expertise; requires multidisciplinary input
**Comprehensive protocols & networks**			
Early MCS, rapid PCI, multidisciplinary shock teams, structured care pathways	NCSI registry; Senman et al.	Associated with markedly lower in-hospital mortality when implemented effectively	Relies on system-level organisation, regionalisation, and experienced personnel; can standardise early care
**Future strategies**			
AI-driven risk prediction (e.g., STOPSHOCK), biomarker subphenotyping, wearable and smart haemodynamic monitoring	Zweck 2023 [126]; STOPSHOCK	Earlier detection, more accurate risk stratification; precision-guided therapy, potentially improving outcomes and reducing unnecessary interventions	Still in development; require prospective validation, data infrastructure, and clinician integration. Promising for shifting care upstream from late-stage intervention to prevention and early recognition; may support personalised treatment algorithms in future networks

## Data Availability

No new data were created or analyzed in this study. Data sharing is not applicable to this article.

## Abbreviations

The following abbreviations are used in this manuscript:

CS	Cardiogenic Shock
CO	Cardiac Output
AMI	Acute Myocardial Infarction
SHOCK	SHould we emergently revascularize Occluded Coronaries for cardiogenic shocK
MCS	Mechanical Circulatory Support
HF	Heart Failure
SCAI	Society for Cardiovascular Angiography and Interventions
ECMO	Extracorporeal Membrane Oxygenation
NHS	National Health Service
AI	Artificial Intelligence
CCCTN	Critical Care Cardiology Trials Network
CSWG	Cardiogenic Shock Working Group
SBP	Systolic Blood Pressure
PCWP	Pulmonary Capillary Wedge Pressure
SV	Stroke Volume
LVEDP	Left Ventricular End-diastolic Pressure
SNS	Sympathetic Nervous System
RAAS	Renin–Angiotensin–Aldosterone System
NA	Noradrenaline
HR	Heart Rate
MVO2	Myocardial Oxygen Demand
BP	Blood Pressure
SIRS	System Inflammatory Response Syndrome
TNF-α	Tumour Necrosis Factor-alpha
IL-6	Interleukin-6
IL-1β	Interleukin-1 beta
NO	Nitric Oxide
ANGII	Angiotensin II
CABG	Coronary Artery Bypass Graft
PCI	Percutaneous Coronary Intervention
IABP	Intra-aortic Balloon Pump
MI	Myocardial Infarction
VSD	Ventricular Septal Defect
CAD	Coronary Artery Disease
AV	Atrio-ventricular
STEMI	ST-segment Elevation Myocardial Infarction
VSR	Ventricular Septal Rupture
MR	Mitral Regurgitation
RCT	Randomised Controlled Trial
NICE	National Institute of Health and Care Excellence
ESC	European Society of Cardiology
DOREMI	Dobutamine compared with Milrinone
ICU	Intensive Care Unit
AHA	American Heart Association
VA-ECMO	Veno-arterial Extracorporeal Membrane Oxygenation
DAPT	Dual Antiplatelet Therapy
NCSI	National Cardiogenic Shock Initiative
ANCHOR	Assessment of ECMO in acute myocardial infarction with Non-reversible Cardiogenic shock to Halt Organ failure and Reduce mortality
PICS	Post-Intensive Care Syndrome
VAD	Ventricular Assist Device
GDMT	Guideline-directed Medical Therapy
3CT	Critical Care Clinical Trialists
DPP-3	Dipeptidyl peptidase-3
PRO	Patient-reported Outcome
HAC	Heart Attack Centre
NEWS	National Early Warning Score
FoCUS	Focused Cardiac Ultrasound
CS-MDT	Cardiogenic Shock Multidisciplinary Team
AHFC	Advanced Heart Failure Centre
OHCA	Out-of-hospital Cardiac Arrest
CAC	Cardiac Arrest Centre
MRA	Mineralocorticoid Receptor Antagonist
ACS	Acute Coronary Syndrome
ML	Machine Learning
CVD	Cardiovascular Disease
